# Disrespect and abuse during facility-based childbirth in southern Mozambique: a cross-sectional study

**DOI:** 10.1186/s12884-019-2532-z

**Published:** 2019-10-22

**Authors:** Anna Galle, Helma Manaharlal, Emidio Cumbane, Joelma Picardo, Sally Griffin, Nafissa Osman, Kristien Roelens, Olivier Degomme

**Affiliations:** 1 International Centre for Reproductive Health, Department of Public Health and Primary Care, Ghent University, Corneel Heymanslaan 10, entrance 75, UZP 114, 9000 Ghent, Belgium; 2 International Centre for Reproductive Health – Mozambique, Rua das Flores no 34, Impasse 1085, /87 Maputo, Mozambique; 3 Faculty of Medicine, Department of Obstetrics/Gynecology, Eduardo Mondlane University, Av. Salvador Allende, 57 Maputo, Mozambique

**Keywords:** Disrespect and abuse, Mozambique, Quality of care, Maternal health, Family planning, Male involvement

## Abstract

**Background:**

Evidence suggests that many women experience mistreatment during childbirth in health facilities across the world, but the magnitude of the problem is unknown. The occurrence of disrespect and abuse (D&A) in maternity care services affects the overall quality of care and may undermine women’s trust in the health system. Studies about the occurrence of disrespect and abuse in Mozambican health facilities are scarce. The aim of this study was to explore the experience of women giving birth in hospital in different settings in Maputo City and Province, Mozambique.

**Methods:**

A cross sectional descriptive survey was conducted between April and June 2018 in the Central Hospital of Maputo (HCM) and district hospitals of Manhiça and Marracuene, Maputo Province, Mozambique. Five hundred seventy-two exit interviews were conducted with women leaving the hospital after delivery. The questionnaire consisted of the following components: socio-demographic characteristics, the occurrence of disrespect and abuse, male involvement during labor and childbirth and intrapartum family planning counselling and provision.

**Results:**

Prevalence of disrespect and abuse ranged from 24% in the central hospital to 80% in the district hospitals. The main types of D&A reported were lack of confidentiality/privacy, being left alone, being shouted at/scolded, and being given a treatment without permission. While very few women’s partners attended the births, the majority of women (73-80%) were in favor of involving their partner as a birth companion. Intrapartum counseling of family planning was very low (9-17%).

**Conclusion:**

The occurrence of disrespect and abuse was much higher in the district hospitals compared to the central hospital, emphasizing the high need for interventions outside Maputo City. Allowing male partners as birth companions should be explored further, as women seem in favor of involving their partners. Investing in intrapartum counselling for family planning is currently a missed opportunity for improving the uptake of contraception in the country.

## Introduction

Maternal mortality refers to deaths caused by complications from pregnancy or delivery. From 1990 to 2015, during the Millennium Development Goals (MDGs) era, the global maternal mortality ratio declined by 44% – from 385 deaths to 216 deaths per 100,000 live births, based on UN inter-agency estimates. Despite the fact that every region has advanced, the maternal mortality ratio is still very high in sub-Saharan Africa compared to the rest of the world [1]. Maternal mortality reduction remains a priority under in the new Sustainable Development Goals (SDGs). By 2030, the global community wants to reduce the global maternal mortality ratio (MMR) to fewer than 70 maternal deaths per 100,000 live births.

Global efforts during the MDGs era have largely focused on increasing antenatal care (ANC) coverage and facility-based childbirth as a key mechanism to reduce maternal mortality [2]. These efforts met with some success. There was much less emphasis on quality of care, although individual studies suggest that poor quality is limiting health gains [3, 4]. Improving quality of care, along with women’s experiences of care, has been highlighted as a key strategy to further reduce preventable maternal mortality and morbidity and achieve the health-related SDG targets by the World Health Organization (WHO) [5]. In 2016, WHO published new guidelines for improving quality of care for mothers and newborns in health facilities, which included an increased focus on respect and preservation of dignity. Experience of care is as important as clinical care provision in achieving the desired person-centred outcomes in the WHO framework for improving quality of care for pregnant women during childbirth [5]. Recent evidence suggests that many women experience mistreatment and are abandoned during childbirth in health facilities across the world, but the magnitude of the problem is unknown [6–10]. An often cited framework for describing interpersonal aspects of care during labor and delivery are the seven domains of disrespect and abuse (D&A) defined in Bowser and Hill’s landscape evidence review, published in 2010: physical abuse; non-consented care; non-confidential care; non-dignified care; discrimination; abandonment of care; and detention in facilities [11]. Afterwards The White Ribbon Alliance spread the Respectful Maternity Care Charter: The Universal Rights of Childbearing Women, a statement grounded in the Universal Declaration of Human Rights [12].

The mistreatment of women during childbirth often occurs at the level of the interaction between women and healthcare providers but deficiencies in the health care system (e.g. lack of adequate personal and poor infrastructure) also contribute to its occurrence [13–15]. The occurrence of disrespect and abuse in maternity care services may undermine women’s trust in the health system and deter them from seeking facility-based care for delivery [16]. Disrespect and abuse during childbirth is more and more being recognized as an indicator of poor quality of care and cited as a key barrier in achieving better maternal health outcomes [17].

Mozambique, with a maternal mortality ratio of 489 maternal deaths per 100 000 livebirths in 2015 and only 54% of births attended by a skilled birth attendant, is one of the priority countries for improving maternal health [18]. Several actions have been taken and progress is ongoing but slow. Recognising the importance of quality of care, since 2007 the MoH (Ministry of Health) of Mozambique has made humanization and patient friendly care during ANC and delivery one of its priorities [11]. Over time, the culture of promoting Respectful Maternity Care (RMC) has become more widespread in Mozambique and the MoH has transformed a selection of maternity wards into centers of quality and humanized Maternal and Newborn Health (MNH) care provision under the “Iniciativa Maternidade Modelo” (Model Maternity Initiative). Respectful maternity care is one of the essential packages of the model and includes respect for beliefs, traditions, and culture; the right to information and privacy; choice of a companion; freedom of movement and position; skin-to-skin contact and early breastfeeding; appropriate use of technology and effective lifesaving interventions; and prevention of violence and disrespectful care [11]. By 2017 the initiative was implemented in all hospitals (central, provincial and district) within the country and almost half of the health centres [unpublished report JHPiego & MoH]. However, no evaluation has been conducted so far from the perspective of users after introducing this model. Studies examining the prevalence of disrespect and abuse in maternity care in Mozambique are scarce, especially in comparison to other countries in the region like Tanzania, South-Africa and Kenya [12, 15, 19, 20]. Recognizing that poor experiences for women might lead to less deliveries in the facilities and affect the quality of care by several pathways, this study aims to assess the experience of women giving birth in hospital in different settings in Maputo City and Province, Mozambique.

## Methods

### Data collection tool

A cross sectional descriptive survey was conducted between April and June 2018 in the Hospital Central de Maputo (HCM) and district hospitals of Manhiça and Marracuene in Maputo Province, Mozambique. HCM is a tertiary referral hospital with on average 20 deliveries a day. HCM is the only hospital in the country equipped to handle advanced operations, thereby serving as the last referral center for the entire country [21]. Manhica and Marracuene district hospital are secondary level hospitals with on average 10 and 5 deliveries a day, respectively. Self-referral and direct access is very common in all three facilities [22]. Exit-interviews were conducted with women leaving the hospital after delivery. The questionnaire consisted of the following components: socio-demographic characteristics, male involvement during labor and childbirth, intrapartum family planning (FP) services and experience of care. A normal delivery was defined as a vaginal delivery without the use of forceps, vacuum extraction or other medical interventions. A vaginal delivery involving a second degree tear or episiotomy was considered as a normal delivery. Experience of care was measured by using 23 verification criteria of disrespect and abuse, subdivided in the 7 categories, according to Bowser and Hill’s landscape evidence review [6, 7, 9, 23]. The questionnaire was translated into Portuguese and can be found in attachment (see Additional file 1). Four female data collectors, not involved in the women’s care, were recruited and received a 1 week training regarding the study procedures, data collection tool and ethical research principles before embarking on data collection. All data collectors were trained to translate the questions from Portuguese to the local dialect (Changana) for participants who did not speak Portuguese.

### Sample size

We wanted to measure the prevalence of disrespect and abuse in hospitals presenting different characteristics – in this case district hospitals and a referral hospital. A single population proportion formula was used to estimate the sample size with assumptions of 5% precision, 95% confidence, and a 10% non-response rate. An assumption that 20% of the women would experience some form of disrespect or abuse was made, based on other studies [24, 25]. The final calculated sample size was 246 for each type of facility (district vs central hospital), which resulted in a total sample size of 592.

### Data collection procedure

We conducted exit interviews with women staying at the maternity unit: all women aged 18–45 years who had delivered at the participating hospitals and who spoke Portuguese or Changana, were invited for an interview. Minors were not included because additional procedures would be required for ethical reasons (e.g. consent of parents, closer follow up).

Data collection continued until the required sample size was reached. Every morning the data collectors visited the post-partum maternity ward and contacted the head nurse to know which women were ready for discharge. These women were approached and invited to participate in the study. Women were invited after the morning round to avoid presence of health care providers. If they consented to participate the interview took place in a private room in the hospital. The questionnaire was set up in Open Data Kit software and tablet computers were used for data collection.

The questionnaire and recruitment procedure were thoroughly pilot-tested prior to data collection. After the pilot test small adaptations were made to the questions to improve comprehensibility.

### Ethical issues

Ethical approval was obtained from the National Health Bioethics Committee of Mozambique, Health Bioethics Committee of Universidade Eduardo Mondlane (UEM), Hospital Central de Maputo (CIBS UEM&HCM/0008-17) and from the Bioethics Committee of Ghent University (EC/2018/1319). All data collectors were trained in data collection procedures and ethical conduct. During the study data collectors were supervised on a daily basis by the principal investigator (AG). Written informed consent was obtained separately for each study participant. All participants were given detailed information about the study and contact details for further information, concerns or questions after participation.

Prior to the start of the study a meeting was organized with the management team of the delivery ward and maternity ward in all study sites to discuss the objective of the study and data collection procedures. Afterwards the management team introduced the study and research team (principal investigator, supervisor and data collectors) to the head nurse of the maternity ward.

### Data analysis

All data was analyzed using the statistical software package R. Simple descriptive analysis was done to explore sociodemographic characteristics of the population. Differences in socio-demographic characteristics by place of delivery (district versus central hospital) were examined using Pearson’s Chi squared test. Disrespect and abuse (D&A) during childbirth were operationalized using the seven categories described in Bowser and Hill’s landscape analysis [6] (see Table 2). In line with global consensus on describing and defining prevalence from the perspective and experience of the woman [4, 7], prevalence of each of D&A category was calculated using the exit interview data. Women who reported experiencing one or more sub-components of D&A were included in the overall prevalence measure.

While previous studies mostly focus on the outcome “experiencing at least one kind of abuse (yes or no)”, we also took into account the number of forms of violence a woman experienced in our analysis. Most women experienced several forms of abuse, which would be masked by using a binary outcome variable for D&A. The sum score of experiencing D&A for each woman was calculated (varying from 0 to 7) and this variable was used as outcome variable in our negative binomial model. Independent variables for our model were chosen based on the hypotheses that women from certain sub-groups (low educational level, single women, young women, women from rural areas) may be more likely to experience and/or report D&A. The reported intercept (often labeled the constant) is the negative binomial regression estimate when all variables in the model are evaluated at zero [26].

## Results

**Fig. 1:**
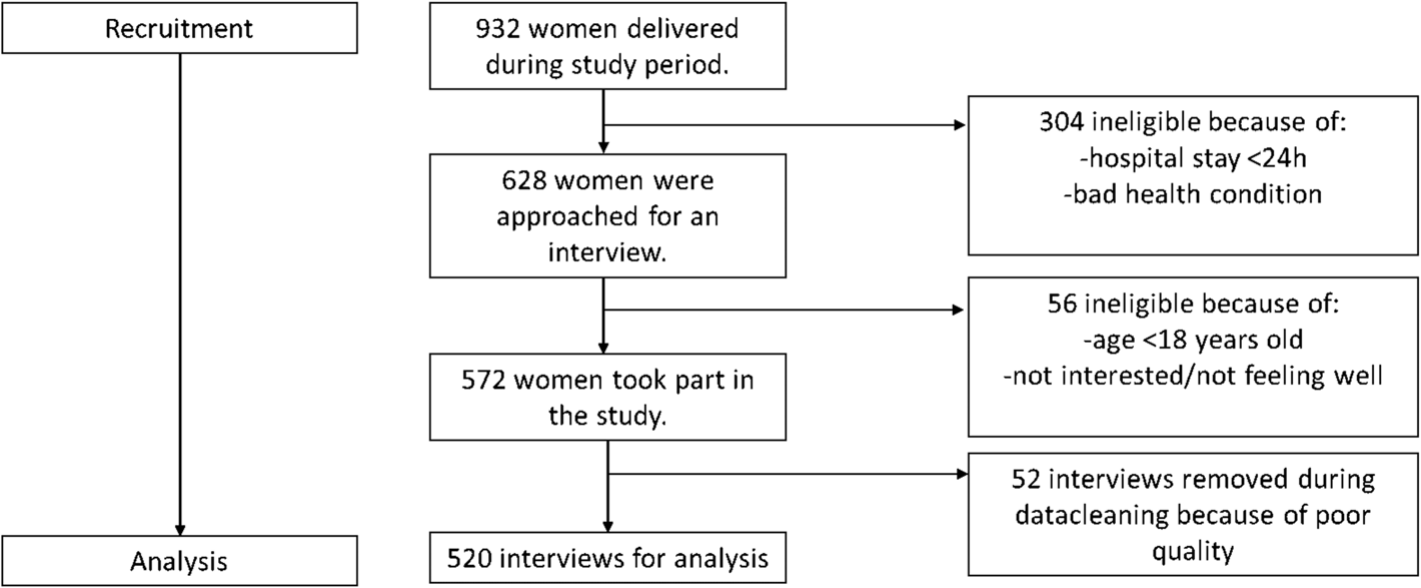
Flow diagram showing process for inclusion in data analysis

In total 932 women gave birth during the study period and 628 women were approached for an interview. The main reasons that some women were not invited to participate were their bad health condition or that they went home very soon after birth (< 24 h). Of the 628 women that were invited for the study, 572 participated. The main reason for not participating when invited was being < 18 years old (*n* = 36); other main reasons were not interested or not feeling well. During data cleaning 52 data entries had to be removed because of poor quality and/or incompleteness, resulting in a final sample of 520 women (see Fig. 1). The final dataset did not contain missing data. Sociodemographic characteristics of the participants can be found in Table 1.

**Table 1:** Sociodemographic characteristics

Site	District Hospitals (*N* = 218)		Central Hospital (*N* = 302)		*p*-value (x^2^ test, df)
Educational level woman**	n	%	n	%	*p* = 4e-27 (*x*^2^ = 126, d.f. = 3)
No education	40	18.35	3	0.01	
Primary school (at least 1 year)	173	79.36	181	59.93	
Secondary school	3	1.38	32	10.60	
Higher education	2	0.92	86	28.48	
Marital Status	n	%	n	*%*	*p* = 0.38 (*x*^2^ = 0.77, d.f. = 2)
Single	48	22.02	61	20.20	
In relationship	167	76.61	241	79.80	
Divorced	3	1.38	0	0	
Educational level partner**	n	%	n	*%*	*p* = 2.8e-29 (x^2^ = 140, d.f. = 4)
No education	7	3.21	1	0	
Primary school (at least 1 year)	135	61.93	132	16.23	
Secondary school	3	1.38	28	9.60	
Higher education	11	5.05	126	41.39	
Don’t know	62	28.44	15	4.97	
Religion**	n	%	n	%	*p* = 4.5e-15 (*x*^2^ = 80, d.f. = 6)
Catholic	25	11.47	74	24.50	
Islam	6	2.75	31	10.26	
Zione	58	26.61	17	5.63	
Protestant	91	41.74	131	43.38	
Independent Christian church	33	15.14	27	8.94	
No religion	3	1.38	0	0	
Others	2	0.92	22	7.29	
Age**	n	%	n	%	*p* = 0.00045 (*x*^2^ = 18, d.f. = 3)
18-21	66	30.28	46	15.23	
> 21-25	48	22.02	71	23.51	
> 25-35	80	36.70	148	49.01	
> 35	24	11.01	37	12.25	
Type of delivery**	n	%	n	*%*	*p* = 7e-10 (*x*^2^ = 42, d.f. = 2)
Normal	194	88.99	195	64.57	
With complications	16	7.34	49	19.21	
Caesarean section	8	3.67	58	16.23	

Levels of significance with the chi-square test:. = *p* < 0.1; * = *p* < 0.05; ** = *p* < 0.01

### Sociodemographic characteristics

In total 145 women participated in the study from the Manhiça district hospital, 73 from Marracuene district hospital and 302 from the central hospital (=HCM). In the central hospital 28.48% of the women completed higher education and 10.60% finished secondary school. In the district hospitals 0.92% completed higher education and 1.38% secondary school. There was a significant difference between women who delivered in the district hospital compared to women who delivered in the central hospital regarding education, education of the partner, religion, age and type of delivery. Overall, women in the central hospital were higher educated, older and had more complicated pregnancies and caesarean sections (see Table 1).

### Experience of care

Of the 302 women interviewed in HCM, 23.51% (*n* = 72) reported at least one kind of abuse or disrespect during labor and/or delivery. In the district hospitals the percentage was significantly higher (X^2^ = 159; d.f. = 1; *p* = 2e-36): 79.82% (*n* = 174) of the women reported at least one form of disrespect or abuse. No significant difference was found in prevalence of disrespect and abuse between the two district hospitals (x^2^ = 0.36; d.f. = 1; *p* = 0.55). Design effect was 0.1904, which is very low (rho = − 0.0054; deff = 0.1904). Between each district hospital and the central hospital the difference in prevalence of D&A was significant as we expected at the start of the study: HCM/Manhiça (x^2^ = 83; d.f. = 1; *p* = 6.6e-20) and HCM/Marracuene (x^2^ = 65; d.f. = 1; *p* = 7.7e-16).

**Fig. 2:**
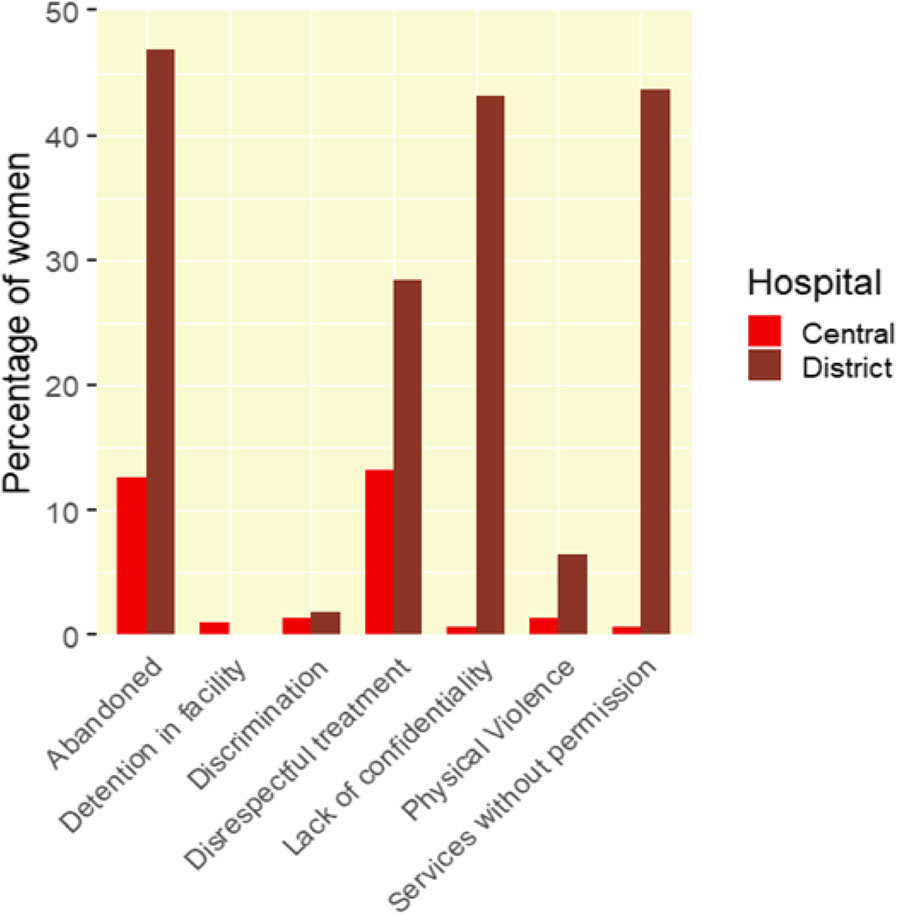
Percentage of women experiencing D&A by category in the district hospitals and central hospital

The provision of non-confidential care (=lack of confidentiality), non-consented care (=services without permission) and abandonment were the most common types of disrespectful care during facility-based childbirth in the district hospital, followed by non-dignified care (=disrespectful treatment) (see Fig. 2). In the central hospital abandonment and non-dignified care were the most prevalent forms of D&A (see Fig. 2). Prevalence of each type of disrespect and abuse can be found in Table 2. Five women mentioned they gave birth alone because nobody came when they called for help (mentioned in category abandoned as “others”). Two women felt disrespected because they had to watch other women giving birth and two women felt disrespected because they had to clean up the bed after delivery (mentioned in category disrespectful treatment as “others”).

**Table 2:** Prevalence different forms of D&A

Site	District Hospital		Central Hospital	
	n	%	n	%
Services without permission				
Caesarean section	1	0.46	1	0.3
Episiotomy	1	0.46	0	0.0
Stitching	14	6.42	0	0.0
Blood transfusion	0	0.00	0	0.0
Sterilization	0	0.00	0	0.0
Injection	82	37.61	0	0.0
Shaving	0	0.00	0	0.0
Others	1	0.46	1	0.3
No	123	56.42	300	99.3
Lack of confidentiality				
Disease (HIV)	1	0.46	0	0.0
Age	3	1.38	0	0.0
Medical history	0	0.00	0	0.0
Absence or position of the father	0	0.00	0	0.0
During labour and delivery	89	40.83	1	0.3
Others	1	0.46	1	0.3
No	124	56.88	300	99.3
Disrespectful treatment				
Threatened with C-section	5	2.29	7	2.3
Scolded, shouted at	57	26.15	30	9.9
Slanderous remarks	6	2.75	2	0.7
Blamed or intimidated	3	1.38	1	0.3
Others	1	0.46	7	0.3
No	156	71.56	262	86.8
Physical Violence				
Beaten, slapped or pinched	0	0.00	0	0.0
Tied down or restrained	0	0.00	0	0.0
Episiotomy sutured without anesthesia	14	6.42	2	0.7
Sexually abused by health worker	0	0.00	0	0.0
Others	0	0.00	2	0.7
No	204	93.58	298	98.7
Discrimination				
Ethnicity	0	0.00	0	0.0
Young and unexperienced	3	1.38	0	0.0
Single motherhood status	0	0.00	0	0.0
HIV sero-positive status	1	0.46	0	0.0
Low socio-economic status	0	0.00	2	0.7
Others	0	0.00	2	0.7
No	214	98.17	298	98.7
Detention in facility				
Unpaid bills mother	0	0.00	1	0.3
Unpaid bills baby	0	0.00	0	0.0
Others	0	0.00	2	0.7
No	218	100.00	299	99.0
Abandoned				
Left alone unattended too often	76	34.86	22	7.3
Denied birth companion	17	7.80	1	0.3
Birth attendant didn’t intervene in urgent situations	0	0.00	1	0.3
Neglected because staff was exhausted	31	14.22	5	1.7
Others	5	2.29	11	3.6
No	116	53.21	264	87.4

### Experience of multiple forms of disrespect and abuse

**Fig. 3:**
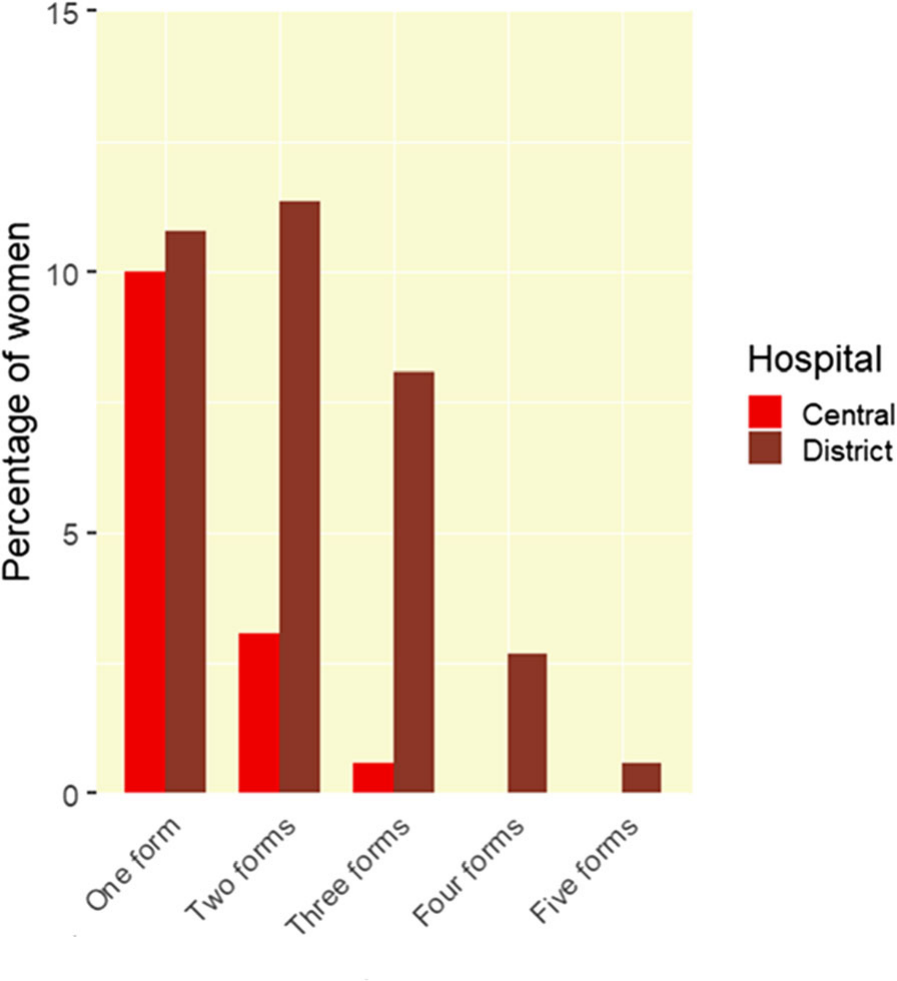
Percentage of women experiencing multiple forms of D&A in the district hospitals and central hospital

The average number of forms of D&A each woman experienced was 1.70 in the district hospital and 0.31 in the central hospital. Women in the district hospitals experienced on average 1.4 more forms of D&A compared to the central hospital, the difference between the two types of site was significant (t = 20, df = 300, *p*-value <2e-16). While women in the central hospital experienced a maximum of 3 forms of D&A, women in the district hospitals experienced a maximum of 5 forms of D&A (see Fig. 3).

We explored which sociodemographic characteristics were associated with experiencing multiple forms of disrespect and abuse by building a binomial negative regression model for both the district hospital and central hospital. Taking into account the AICs (Akaike Information Criterion )[27], a model was selected with the number of forms of disrespect (varying from 0 to 7) as outcome variable and age, marital status, type of delivery, educational level and parity as predictors. Religion and educational level of the husband were also included as covariates but eliminated during model selection as these sociodemographic characteristics were not significant and reduced validity of the model. Table 3 shows the descriptive statistics of the explanatory variables (=predictors).

**Table 3:** Descriptive statistics explanatory variables (predictors)

	Number of forms of violence			
	District Hospitals		Central Hospital	
	Mean	SD	Mean	SD
Educational level				
Primary level	1.72	1.25	0.22	0.50
Secondary or more	1.17	0.98	0.44	0.74
Number or pregnancies				
Primigravida	2.02	1.38	0.41	0.70
Multigravida	1.61	1.20	0.27	0.57
Type of delivery				
Normal delivery	1.76	1.22	0.27	0.61
Delivery with complications	1.62	1.45	0.47	0.68
Cesarean section	0.50	0.76	0.31	0.57
Civil state				
Single	1.7	1.13	0.31	0.62
In relationship	1.69	1.28	0.30	0.61
Age				
< =21 years	1.71	1.33	0.48	0.78
> 21 and = < 25 years	1.88	1.25	0.34	0.63
> 25 and = < 35 years	1.57	1.17	0.26	0.56
> 35 years	1.75	1.29	0.22	0.53

In the district hospital having a caesarean section decreased the number of disrespect with 1.26 (see Table 4). In the central hospital (see Table 5) having a delivery with complications increased the number of D&A with 0.65. Also having completed primary education increased the number of D&A with 0.80. Also age was a significant predictor, younger women experienced significantly more D& A. Every year older decreased the number of D&A with 0.05 (see Table 5).

**Table 4:** Binomial negative regression model D&A in District hospitals

	Estimate	Std. Error	*z*-value	*p*
Effect:				
*Intercept*	0.55	0.15	3.58	0.00034 **
*Number of pregnancies*	−0.01	0.03	− 0.23	0.82
*Having a C-section*	−1.26	0.50	−2.51	0.01*
*Having delivery with complications*	−0.06	0.20	−0.31	0.76
*Having completed primary school*	0.42	0.38	−1.09	0.27
*Being Single*	0.01	0.13	0.12	0.91
*Age*	0.00	0.01	0.31	0.76

Levels of significance:. = *p* < 0.1; * = *p* < 0.05; ** = *p* < 0.01

**Table 5:** Binomial negative regression model D&A in central hospital

	Estimate	Std. Error	z-value	*p*
Effect:				
*Intercept*	− 0.12	0.60	−0.19	0.85
*Number of pregnancies*	−0.03	0.12	−0.26	0.80
*Having a C-section*	0.23	0.30	0.77	0.44
*Having delivery with complications*	0.65	0.28	2.34	0.02*
*Having completed primary school*	0.80	0.23	3.41	0.00064**
*Being Single*	−0.32	0.29	−1.10	0.27
*Age*	−0.05	0.30	−1.09	0.04*

Levels of significance:. = *p* < 0.1; * = *p* < 0.05; ** = *p* < 0.01

### Role of the partner

One man was present during labor and delivery in Marracuene district hospital, and no men were present in Manhiça. In HCM no men were present, this is officially not permitted in this hospital.

Women were asked if they would like to have their husband as their companion during labor and/or delivery (if allowed). The results showed that 79.47% (*n* = 240) of the women in HCM would like their husband to be present and 62.84% (*n* = 137) of the women in the district hospitals. The women were also asked if they thought their husbands would be willing to be their companion, 72.85% (*n* = 220) of the women in HCM and 41.74% (*n* = 91) of the women in the district hospitals believed their husbands would like to accompany them.

### Family planning in the immediate postpartum

Family planning was discussed by the provider with 8.94% (*n* = 27) of the women during their stay in HCM and with 15.60% (*n* = 34) of the women in the district hospitals. Of the women in HCM 0.99% (n = 3) received a contraceptive method. In the district hospitals 1.83% (*n* = 4) of the women received a contraceptive method. Which methods were discussed and provided can be found in Table 6.

**Table 6:** Family planning methods

Site	District Hospitals		Central Hospital	
	n	%	n	%
Methods discussed				
Female condom	14	6.42	19	6.29
Male condom	12	5.50	18	5.96
Lactation amenorrhea Method	0	0.00	1	0.33
Oral contraceptives	29	13.30	13	4.30
Injectable contraceptives	24	11.01	11	3.64
IUD	16	7.34	18	5.96
Implant	26	11.93	24	7.95
Sterilisation	0	0.00	7	2.32
Others	1	0.46	2	0.66
Methods received				
Female condom	1	0.46	0	0
Male condom	1	0.46	0	0
Breastfeeding	0	0.00	0	0
Oral contraceptives	0	0.00	1	0.33
Injectable contraceptives	0	0.00	0	0
IUD	0	0.00	0	0
Implant	1	0.46	0	0
Sterilisation	1	0.46	2	0.66

## Discussion

The prevalence of disrespect and abuse in our study was similar to the prevalence in other countries in the region: 23.51% in the central hospital and 79.82% in the district hospitals. Studies from Ethiopia, Kenya and Tanzania report D&A prevalence rates between 20 and 70% [9, 12, 17, 20]. However, it may be problematic to focus only on overall prevalence of D&A as an outcome, as this covers a wide range of forms of D&A that are very different in nature (e.g. injections without permission versus slapping and beating). In this study we found that more severe forms of abuse such as detention in the facility (for failure of paying) and physical violence (such as slapping) are almost non-existent in the study sites in Mozambique, while studies conducted in other countries often report much higher figures. For example, a systematic review of D&A in Ethiopia estimated a prevalence of 13% for physical abuse and 3.2% for detention in the facility [28]. The implementation of the “Iniciativa Maternidade Modelo” might have contributed to this positive result in Maputo City and Maputo Province and further efforts should focus on reducing abandonment (when the patient is being left alone) and disrespectful treatment (being shouted/scolded at), which continue to be prevalent.

The occurrence of D&A in maternity care services is often considered as a marker for quality of care: it might affect quality of care in both terms of discouraging women to deliver in facilities but also directly through inadequate monitoring during childbirth (eg. infrequent fetal monitoring during labour and delivery, or absence of a skilled provider for resuscitation of the newborn or to intervene in case of bleeding of the mother) [14]. Several participants in our study reported they delivered alone in the health facility, which imposes a serious risk on both mother and child. This might also indicate that the number of women delivering without a skilled birth attendant is probably under reported in the region. Mozambique is struggling with a weak health system, characterized by poor health infrastructure, shortage of providers and insufficient supervision [29]. Certain forms of D&A (abandonment and lack of privacy) we found to be common might be triggered or worsened by resource scarcity within the health system. The inadequate health system resources (lack of separate rooms, insufficient skilled providers) are probably a major contributing factor to certain forms of D&A and prevention should be oriented at this level.

Stigmatization and emotional abuse of women by providers (discrimination of primigravidas due to being unexperienced, slanderous remarks, lack of privacy regarding age) are also a prevalent problem in maternity care in Mozambique, according to our results. Discrimination and stigmatization of certain subgroups in health care settings have been studied mostly in high income countries. The problem has much less been studied in low income countries and has had a strong focus on minority groups and HIV stigmatization [30, 31]. The role of medical education (e.g. training to shape the attitudes of providers) in prevention of discrimination in health care settings may be well recognized, especially in high-income countries, but it is inadequately explored in the context of D&A [10, 32]. On a global level, countries with strong colonial roots often have a health system culture where providers morally instruct and educate their patients [14], which might contribute to the occurrence of D&A in Mozambique. This is in line with research that suggest that nurses’ and midwives’ inferiority in medical hierarchy and lack of power within their own professional and organizational structures might contribute to their need to dominate and control even more disempowered patients [13, 33]. When designing interventions to prevent D&A, a participatory approach with providers will be needed to explore the roots of their abusive behaviors towards women and identify ways to overcome them.

The overall prevalence of D&A in the district hospitals was much higher compared to the central hospital (79.8% vs 23.5%). Furthermore, we could demonstrate that women in the district hospitals more often experience a combination of different forms of disrespect and abuse compared to the central hospital. In our study the lower D&A prevalence in the central hospital compared to the district hospitals might be related to the fact that providers work under better circumstances in the central hospital. The central hospital is a teaching hospital with more supervision and control mechanisms than the district hospitals (e.g. extensive maternal death audits and academic meetings), and in general the maternity care system in Maputo City is better resourced than the rest of the country [34].

There is no consensus in the literature on the role sociodemographic and institutional factors play in the actual prevalence or reporting of D&A [9, 13, 19, 35]. Moreover, the influence of these factors might be very context specific [10]. This was confirmed in our study: sociodemographic factors played a different role in the central hospital compared to the district hospitals. In our study women with a secondary degree experience and/or report more forms of D&A [12]. This relationship might be related to the fact that these women expect higher standards of care and more easily recognize abusive behavior [12, 36]. Echoing the results of other studies, women in our study who had a delivery with complications reported more D&A [37]. Age was a protective factor against D&A in the central hospital. Several qualitative studies report that especially young and unexperienced women experience D&A due to power dynamics and low status [38, 39]. But they might also less easily recognize and report unacceptable behavior of providers, which might explain the contradicting findings in the literature. Nevertheless, more qualitative data from both women and providers will be needed to explore contributing factors regarding D&A in the Mozambican health system and specific context.

Labor companionship is a key component of providing respectful maternity care and has been included as one of the WHO standards for improving the quality of maternal and newborn care in health facilities [40]. Despite the benefits of a companion of choice throughout labor, implementation of this approach is not universal [41]. In Mozambique all maternities are officially obliged to allow birth companions since the introduction of the Model Maternity Initiative in 2017. However, in practice there are different rules depending on the provider (e.g. only women are allowed, no traditional birth attendants, only during the day, not able to switch) [experience in the field]. In most facilities in Mozambique it is strictly forbidden to allow male partners as birth companions during labor and delivery. This rule is partly linked with an overall lack of privacy on maternity wards (e.g. women deliver in beds next to each other in one room), which is perceived as more problematic when men are allowed to be present. However, as public facilities are improving more maternities now have separate rooms, and also in very small facilities privacy can often be guaranteed due to low numbers of births. Recognizing that the Respectful Maternity Care Charter and MoH policy state that women have the right to choose their own birth companion it is then contradictory to only allow female birth companions [42]. Also the World Health Organization recommends in their intrapartum guidelines that a parturient woman should be encouraged to have a supportive companion she trusts and can feel at ease with in labor and birth [5, 43].

Our study found that a majority of women were in favor of involving their male partner as birth companion and many also believe their partners would be in favor. The desire of women to involve their male partner should be taken into consideration by maternities and might be a motive to reconsider current restrictions, where privacy can be guaranteed. Another argument for allowing men on maternity wards is that research suggests that disrespectful care would be less frequent if partners were present [44–46]. Birth companions in general are a protective factor against D&A [13, 19], and there is some evidence that bringing in the male partner might further protect the women against experiencing D&A. A study from Tanzania showed that male partners of women who experience abuse during labor or delivery find it easier to request better care or lodge a complaint than the women themselves [46]. Qualitative studies on experiences of men who have attended the births of their children in Malawi also showed that with a supportive environment and positive attitude of the midwives, it is possible to involve male partners during childbirth and for this to be a positive experience for both men and women [47, 48]. Further research is needed to explore the feasibility of allowing men in the delivery room in Mozambique and to examine potential strategies that create the ideal conditions for men to be present during labor and birth as the birth companion. It would also be interesting to examine whether involving men in maternity care might have an impact on the prevalence of disrespect and abuse during childbirth.

Offering modern contraception services as part of care provided during childbirth increases postpartum contraceptive use and is likely to reduce both unintended pregnancies and pregnancies that are too closely spaced [49]. It is recommended by the WHO standards for improving the quality of maternal and newborn care in health facilities [40] but very often neglected in studies examining quality of childbirth care [50]. Our study showed that both in the district hospitals and the central hospital the number of women receiving counselling about family planning was very low (17 and 9% respectively). For women with limited access to health care in facilities, delivery at a facility affords a unique opportunity to address their fertility intentions and need for contraception: it does not require a return visit that may be prohibitively expensive or inconvenient. Previous studies have shown that in the year following childbirth, many women want to postpone or avoid further births, but do not use a contraceptive method [51]. Offering family planning counselling before women leave the hospital might be an important and unique opportunity to protect women from an unplanned pregnancy, as only a minority of women (40-44%) return to the health facility for a postnatal care visit in Mozambique [18, 52]. Evidence has shown that discussing family planning before discharge from the maternity ward is an effective intervention to increase the uptake of family planning methods postpartum [53–56]. Mozambique has a comprehensive strategy to reduce the unmet need for family planning including guidelines for integrating family planning counselling and provision of contraceptives across the health service including during the intrapartum period [57–60]. However, increased attention is required to translate this policy into practice in order to improve uptake of family planning services in the post-partum period.

## Limitations

Currently there is a lack of standardized definitions, instruments, and study methods to quantify D&A in childbirth facilities, which affects the generalizability and comparability of results [7]. A validated instrument, taking into account the severity of each form of abuse, is needed if we want to continue to compare overall prevalence of D&A across different countries and/or regions. Furthermore some reported forms of D&A might not actually constitute mistreatment: for example, giving an injection without permission or stitching a first degree tear without anesthesia might be justified under certain medical conditions. A recent qualitative evidence synthesis also showed that RMC is a broader concept than merely the absence of mistreatment, although the two are intertwined [61]. While qualitative studies show that provider’s and women’s views on respectful maternity care are widely consistent globally, further research is needed to assess the validity and responsiveness of quantitative indicators to measure RMC [61].

Previous studies have shown that the factors that contribute to D&A in maternity care services and potential prevention measures are very context specific, which was confirmed in our study. We acknowledge that our study results cannot be generalized to other settings and further studies in different contexts in Mozambique are needed. Nevertheless we were able to show that D&A is a prominent problem in the country despite some significant quality improvement programs in maternity care over the last years.

We did not include minors, which is a limitation of our study. Data suggest that D&A and especially discrimination happen more often with adolescents, and our study found that increased age was protective against D&A. A follow-up study focusing on the occurrence of D&A in this specific group is recommended. In addition, our study was conducted inside the health institution, where social desirability bias can underestimate the occurrence of D&A. A community-based study might give women more freedom to express their feelings and report their experiences without fear, and eliminate this social desirability bias [62].

## Conclusions

The overall prevalence of disrespect and abuse in our study was similar to the prevalence in other countries in the region but the more severe forms of abuse such as detention in the facility (for failure of paying) and physical violence (such as slapping) are almost non-existent. Occurrence of disrespect and abuse was much higher in the district hospitals. The majority of women were in favor of involving their male partner as birth companion and further research is needed to explore the feasibility of allowing men in the delivery room. Both in the district hospitals and the central hospital the number of women receiving counselling about family planning was very low. Investing in intrapartum counselling for family planning is currently a missed opportunity for improving the uptake of contraception in the country.

## Supplementary information

**Additional file 1.** Questionnaire.

## Data Availability

The datasets used and/or analyzed during the current study are available from the corresponding author upon reasonable request.

## Abbreviations

ANC: Antenatal Care
D&A: Disrespect and Abuse
FP: Family Planning
HCM: Hospital Central de Maputo
MCHIP: Maternal and Child Health Integrated Program
MDGs: Millennium Development Goals
MMR: Maternal Mortality Ratio
MNH: Maternal and Newborn Health
MoH: Ministry of Health
RMC: Respectful Maternity Care
SDGs: Sustainable Development Goals
UEM: Universidade Eduardo Mondlane
WHO: World Health Organization
