# Supplementary material for: Disrespect and abuse during facility-based childbirth in southern Mozambique: a cross-sectional study
Source: BMC Pregnancy Childbirth. 2019 Oct 22;19:369. doi: 10.1186/s12884-019-2532-z (PMC6805678; doi:10.1186/s12884-019-2532-z)
Supplement: Supplementary file 1 — Additional file 1. Questionnaire. [file 12884_2019_2532_MOESM1_ESM.docx]

Additional File 1: Questionnaire

**Código do Questionário ____/____/____/____/**

**A. Aspectos de Identificação Questionário**

**1. Início da entrevista /___/___/ : /____/____/ (hh/mm)**

**2. Nome do inquiridor /____________________/**

**3. Data /___/___ /____/___ /___/____/____/____/ (dia/mês/ano)**

**4. Hospital/Centro de Saúde**

(1) Manhiça Sede

(2) Marracuene Sede

(3) HCM

**B. Dados Socio-Demográficos do participante**

1. **Idade /___/____ / (anos completos fixar o intervalo de 18a 50 anos)**

**Se não sabe, digite 99**

1. **Nivel de escolaridade**

(1) Nenhum

(2) Alfabetização (sabe ler e escrever)

(3) EP1 (1ª a 5ª classe)

(4) EP2 (6ª a 7ª classe)

(5) ESG1 (8ª a 10ª classe)

(6) ESG2 (11ª a 12ª classe)

(7) Ensino Básico Profissional

(8) Ensino Médio Profissional

(9) Ensino Superior

**3. Estado civil**

(1) Solteira

(2) Casada /União marital monogâmica

(3) União marital poligâmica

(4) Divorciada / Separada

(5) Viúva

**4. Nivel de escolaridade do parceiro**

(1) Nenhum

(2) Alfabetização (sabe ler e escrever)

(3) EP1 (1ª a 5ª classe)

(4) EP2 (6ª a 7ª classe)

(5) ESG1 (8ª a 10ª classe)

(6) ESG2 (11ª a 12ª classe)

(7) Ensino Básico Profissional

(8) Ensino Médio Profissional

(9) Ensino Superior

(10) Não sabe

**5. Religião.**

(1) Católica

(2) Islâmica

(3) Sião/Zione

(4) Protestante/Evangélica

(5) Cristã independente

(6) Pagã/Nenhuma

(7) Outra, Especifique/_________________________________ /

**6. Quantas vezes você ficou gravida, inclua os casos de abortos ou perda de gravidez /__/__/ (Até 20 gravidezes)**

**7.** Como foi o último parto?

(1) Normal s/ complicações

(2) Cesariana

(3) Normal c/ complicações

(4) Não sabe

**C. ENVOLVIMENTO MASCULINO NOS SERVIÇOS DE SAÚDE**

1. **Se fosse permitido, gostaria que o seu marido lhe acompanhasse a sala de parto?**

(1) Sim

(2) Não

1. **Acha que seu marido gostaria de ser seu acompanhante?**

(1) Sim

(2) Não

1. **Durante a sua estadia no hospital alguém conversou consigo sobre planeamento familiar no pós-parto?**

(1) Sim

(2) Não (se não, passa para pergunta 5)

Se sim:

**4. Qual método de planeamento familiar foi mencionado? (escolha múltipla)**

(1). Preservativo masculino

(2). Preservativo feminino

(3). Método de lactação (amamentação)

(4). Contraceptivos orais (Microgynon/Microlut()

(5) Depoprovera (injecção)

(6). DIU (aparelho)

(7). Implante

(8). Esterilização (laqueação)

(9). Outros: Especifique__________________

**5. Você recebeu um método?**

(1) Sim

(2) Não (se não, passa para pergunta 7)

Se sim:

**6. Recebeu preservativos? (escolha múltipla)**

(1). Preservativo masculino (Aceita a opção 2)(2). Preservativo feminino (Aceita a opção 1)

(3) Não (nega as opções 1 e 2)

6.1. Recebeu um outro método? (Única opção)

(1) Método de lactação (amamentação)

(2). Contraceptivos orais (Microgynon/Microlut/…)

(3) Depoprovera (injecção)

(4). DIU (aparelho)

(5). Implante

(6). Esterilização

(7) Não

**7. Conversou com seu marido sobre o planeamento familiar no pós-parto?**

(1). Sim (Passa para a 9)

(2). Não

8. Pretende conversar com o seu marido/parceiro sobre o PF no pós-parto?

(1). Sim

(2). Não

**9. O provedor conversou com seu marido sobre o planeamento familiar no pós-parto?**

(1). Sim

(2). Não

**D. DESPESAS DURANTE O PARTO**

**1. Você pagou algum dinheiro para o transporte para se deslocar até aqui (unidade sanitária) ?**

**(1). Sim**

**(2). Não** **(Se não, passa para pergunta 3)**

**2. Quanto pagou? : ..... (MZN) (fixar em 4 digitos, até 5000 Mt)**

Gostaríamos de saber se pagou algum dinheiro para o parto e medicamentos usados durante o parto. É importante para o estudo ter informação sobre todas as cobranças feitas.

Algumas pessoas pagam mais do que a tarifa indicada—como gratificação ou porque o profissional de saúde pediu. Por favor, também considere este tipo de pagamento nas próximas perguntas.

3. Durante a sua estadia no hospital teve que pagar por algum serviço recebido?

(1) Sim

(2) Não (se não, passa para pergunta 22)

4.Pagou pelo parto?

(1) Sim

(2) Não **( Se não passa para pergunta 6)**

5.Quanto pagou pelo parto: ..... (MZN) (Fixar em 5 digitos, até 25000 Mt)

6. Pagou pelas análises / testes?

(1) Sim

(2) Não **( Se não passa para pergunta 8)**

7. Quanto pagou pelasanalise/testes: ..... (MZN) (fixar e 4 digitos, 5000 Mt)

8. Pagou pela injecção? (Normalmente antes ou depois do parto da-se uma injecção)

(1) Sim

(2) Não **( Se não passa para pergunta 10)**

9. Quanto pagou pela injecção: ..... (MT) (fixar em 4 digitos, até 5000 Mt)

10. Pagou pelos medicamentos?

(1) Sim

(2) Não **( Se não passa para pergunta 12)**

11. Quanto pagou pelos medicamentos: ..... (MZN) (fixar em 4 digitos, até 5000 Mt)

12. Pagou pelo material usado?

(1) Sim

(2) Não **( Se não passa para pergunta 14)**

13. Quanto pagou pelo material usado: ..... (MZN) (fixar em 4 digitos, até 5000 Mt)

14 Fez algum pagamento extra para ter atendimento especial?

(1) Sim

(2) Não **( Se não passa para pergunta 16)**

15. Quanto foi o pagamento extra para ter atendimento especial ..... (MZN) (fixar em 4 digitos, até 5000 Mt)

16.Deu alguma gratificação ou pagamento informal ao provedor de saúde?

(1) Sim

(2) Não **( Se não passa para pergunta 18)**

17. Quanto pagou de gratificação ou pagamento informal ao provedor de saúde: ..... (MZN) (fixar em 4 digitos, até 5000 Mt)

18. Fez algum outro tipo de pagamento?

(1) Sim

(2) Não **(Se não passa para pergunta 20)**

19. Quanto foi : ..... (MZN)? (fixar em 4 digitos, até 5000 Mt)

20.Se tiver pago alguma gratificação, de quem foi a iniciativa (ÚNICA OPÇÃO DE RESPOSTA)

a) (1) Minha iniciativa

1. (2) Trabalhador pediu
2. (3) Sempre se paga
3. (4) Outro: Especifique :_______________________________

21. Se tiver pago alguma gratificação, qual foi o principal motivo do pagamento: (ÚNICA OPÇÃO DE RESPOSTA)

1. (1) Ser atendido mais rápido
2. (2) Ser atendido por uma pessoa em particular
3. (3) Ter um parto mais rápido
4. (4) Ter melhor serviço
5. (5) Outro: Especifique ______________________________

22. Se não tiver pago pelos serviços recebidos, porque é que não pagou?

(1) Este serviço nunca é pago

(2) Conheço o pessoal

(3) Tenho direito a cuidados gratuitos

(4) Pagarei mais tarde

(5) Já paguei antes (continuação de tratamento)

(6) Outro, especifique: ____________

**E. EXPERIÊNCIA DE CUIDADOS**

Mencione tudo o que aconteceu durante seu último parto **(escolha múltipla):**

**1. Quais dos seguintes serviços foram oferecidos sem o seu consentimento ou permissão (Ler todas opções de resposta):**

**(Ler tudo)**

(1). Cesariana (operação)

(2). Episiotomia (corte entre vagina e o ânus para permitir a passagem do bebe durante o parto)

(3). Transfusão de sangue

(4). Esterilização

(5). Injeção para a diminuição do tempo de parto (o pode ser depois)

(6). Corte dos pelos púbicos

(7) Nenhum serviço

(8). Outros: Especifique ___________________

**2. Houve falta de confidencialidade ou divulgação de informações sem a sua permissão (Ler todas opções de resposta):**

(1). De algum doenca (por ejemplo estado de HIV)

(2). Idade

(3). Historial médico

(4) Paternidade do seu filho

(5) Falta de privacidade durante trabalho de parto

(6) Não

(7). Outros: Especifique ___________

**3. Teve um atendimento não dignificante ou desrespeitoso que incluem (Ler todas opções de resposta):**

(1). Foi ameaçada com uma cesariana para que parasse de gritar ou andar

(2). Zangaram ou berraram consigo durante o trabalho de parto

(3). Foi insultada pelas parteiras/enfermeiras durante o parto

(4). Foi culpada durante o parto por ter um parto difícil ou pela dor

(5) Não

(6). Outros: Especifique __________________

**4. Abusos físicos durante o parto (Ler todas opções de resposta):**

(1). Foi espancada, esbofeteada ou beliscada

(2). Foi amarrada ou confinada durante o parto

(3). Suturaram episiotomia sem anestesia

(4). Foi abusada sexualmente (inclui ser tocada nas partes intimas do corpo sem seu consentimento) pelo trabalhador de saúde durante o trabalho de parto

(5) Não

(6). Outros: Especifique

**5. Foi discriminada durante o trabalho de parto com base em (Ler todas opções de resposta):**

(1). Etnia

(2). Juventude e inexperiência

(3). Estar sem parceiro

(4). Estatuto de seropositividade para HIV

(5). Baixo estatuto socio-económico

(6) Não

(7). Outros: Especifique ____________________

**6. Durante o parto sentiu-se abandonada ou negligenciada da seguinte forma (Ler todas opções de resposta):**

(1). Foi abandonada e atendida na segunda fase do trabalho do parto (fase de expulsão do bebe)

(2). Foi recusado o acompanhamento pelo marido ou parentes próximos durante o trabalho de parto

(3). Em situações de ameaça de vida (ou complicações no parto) a parteira/enfermeira da maternidade falhou na intervenção ou pedido de ajuda da equipa mais experiente

(4) Não recebeu atenção solicitada porque o pessoal estava cansado

(5) Não

6. 0utros: Especifique

**7. Foi retida ou mantida mais tempo do que necessário na unidade sanitária por seguintes motivos (Ler todas opções de resposta):**

(1). Não pagou a conta de hospital até receber a alta

(2) Não pagou a conta do seu filho após a alta

(3) Não

(4). Outros: Especifique ____________________

**8. Tem alguma sugestão para melhorar os serviços** (pPergunta aberta)

**__________________________________________________________________________________**

10. Observações do inquiridora

__________________________________________________________________________________

**Fim da entrevista**
